# Dementia as a presentation of motor neurone disease: a case report

**DOI:** 10.1192/j.eurpsy.2023.1585

**Published:** 2023-07-19

**Authors:** A. S. C. Miranda, I. Franco, J. Miranda

**Affiliations:** Psychiatry, Centro Hospitalar de Leiria, Leiria, Portugal

## Abstract

**Introduction:**

Amyotrophic lateral sclerosis (ALS), the most common disease within motor neuron diseases (MND), and frontotemporal dementia (FTD) belong to a broad spectrum of neurodegenerative diseases that are sometimes clinically overlapping.

**Objectives:**

The aim of the description of this case report is to sensitize professionals to this type of presentation and encourage the investigation of signs and symptoms of motor decline in patients with suspected FTD.

**Methods:**

Research in the patient’s clinical process. Framing the clinical case in the current literature, searching the terms “frontotemporal dementia” and “amyotrophic lateral sclerosis” in the Pubmed database.

**Results:**

A 71-year-old patient, followed in psychiatry for several years for Dysthymic Disorder. At the end of 2020, he presented cognitive and behavioral changes, with rapid progression, with a marked loss of functionality, compatible with dementia. In 2021, it was noticed a motor decline, which progressively worsened. In this sense, an electromyographic study of the limbs was performed with an abnormal result, compatible with a diagnosis of Motor Neuron Disease.

**Conclusions:**

A significant overlap of these two disorders has been observed clinically. Thus, the presentation of a patient with dementia, specifically suspected of having FTD, should ring the bell to the presence of signs and symptoms of motor impairment.

Several studies have been carried out in order to understand the relationship between these entities, and the discussion remains whether their presentation together constitutes or not a form of phenotypic presentation of ALS.

**Disclosure of Interest:**

None Declared

